# Supplementary material for: Modelling the Reallocation of Time Spent Sitting into Physical Activity: Isotemporal Substitution vs. Compositional Isotemporal Substitution
Source: Int J Environ Res Public Health. 2021 Jun 8;18(12):6210. doi: 10.3390/ijerph18126210 (PMC8229040; doi:10.3390/ijerph18126210)
Supplement: Supplementary file 1 [file ijerph-18-06210-s001.zip › ijerph-1179195-SI.pdf]

Supplementary Table S1. The associations of reallocating time spent sitting with standing and with stepping.

	Compositional isotemporal substitution	
	Sitting to Standing	Sitting to Stepping
BMI	**−0.05 (−0.08; −0.02)**	**−0.33 (−0.40; −0.27)**
Waist circumference	**−0.07 (−0.10; −0.04)**	**−0.30 (−0.36; −0.23)**
Cholesterol	−0.01 (−0.04; 0.02)	0.03 (−0.03; 0.10)
HDL Cholesterol	**0.05 (0.01; 0.08)**	**0.17 (0.10; 0.23)**
LDL Cholesterol	−0.01 (−0.05; 0.02)	0.03 (−0.04; 0.09)
Triglycerides	**−0.06 (−0.09; −0.03)**	**−0.17 (−0.24; −0.10)**
HbA1c	0.02 (−0.01; 0.06)	0.00 (−0.07; 0.07)
CMR Risk Score	**−0.03 (−0.05; −0.01)**	**−0.13 (0.17; −0.10)**
CMR Risk Score (without adiposity)	**−0.02 (−0.04; 0.00)**	**−0.09 (−0.12; −0.05)**
	Traditional isotemporal substitution	
	Sitting to Standing	Sitting to Stepping
BMI	**−0.07 (−0.11; −0.04)**	**−0.38 (−0.46; −0.30)**
Waist circumference	**−0.08 (−0.12; −0.05)**	**−0.35 (−0.43; −0.27)**
Cholesterol	−0.01 (−0.05; −0.02)	0.04 (−0.04; 0.12)
HDL Cholesterol	**0.05 (0.01; 0.08)**	**0.21 (0.13; 0.29)**
LDL Cholesterol	−0.01 (−0.04; 0.02)	0.02 (−0.06; 0.10)
Triglycerides	**−0.06 (−0.10; −0.03)**	**−0.20 (−0.29; −0.12)**
HbA1c	0.02 (−0.02; 0.06)	0.00 (−0.09; 0.08)
CMR Risk Score	**−0.03 (−0.05; −0.02)**	**−0.16 (−0.21; −0.12)**
CMR Risk Score (without adiposity)	**−0.02 (−0.04; 0.00)**	**−0.11 (−0.16; −0.07)**

* All variables used in the analyses have been standardised by calculating z scores. Bold values represent significant associations set at an alpha of ≤0.05.
